# Editorial: Cellular immunotherapy: transforming cancer treatment

**DOI:** 10.3389/fimmu.2025.1724025

**Published:** 2025-10-29

**Authors:** Yahia Moualla, Laura Belver

**Affiliations:** ^1^ Josep Carreras Leukemia Research Institute (IJC), Badalona, Barcelona, Spain; ^2^ Program Against Cancer Therapeutics Resistance (ProCURE), Catalan Institute of Oncology (ICO), Badalona, Barcelona, Spain

**Keywords:** cell-based immunotherapies, CAR (chimeric antigen receptor), TIL (tumor infiltrating lymphocytes), CIK (cytokine-induced killer) cells, engineered TCR T cells

Cell-based cancer therapies first emerged in the 1950s with the pioneering work of Prof. Edward Donnall Thomas, who established bone marrow transplantation as a novel and groundbreaking therapeutic strategy for hematologic malignancies. Beyond achieving cures in patients who previously had minimal prospects for survival, this therapy, which remains a standard of care for leukemia and lymphoma, transformed oncology by introducing a paradigm shift: employing the body’s own immune cells to combat cancer (1). Building on this breakthrough, significant milestones have been achieved over the following decades with the development of therapies that harness the immune system to treat malignancies.

Currently, cell-based immunotherapies can be broadly classified into two main categories: those using native immune cells, which are typically isolated from the patient and subsequently expanded, activated and/or modified *ex vivo* before reinfusion, and those employing genetically engineered cells that express synthetic receptors to enhance target specificity. Within the first category, therapies employing dendritic cell (DC) vaccines, tumor-infiltrating lymphocytes (TILs), cytokine-induced killer (CIK) cells, and natural killer (NK) cells are under active evaluation across various clinical settings (2–4). The second category encompasses strategies based on engineered tumor-specific T cell receptors (TCR) and chimeric antigen receptors (CAR), the latter applied not only to T cells (CAR-T), but also to NK cells (CAR-NK) and macrophages (CAR-M) (5). Among these, only a subset of CAR-T cell therapies, along with one DC vaccine and one TIL-based therapy have so far received regulatory approval.

The groundbreaking discovery of DCs by Prof. Ralph Steinman and Prof. Zanvil Cohn in the 1970s laid the scientific foundation for the development of DC vaccines (6). Exploiting the antigen-presenting capabilities of these cells, DCs are loaded with tumor-specific antigens *ex vivo* and then administered to the patient to elicit a targeted anti-tumor immune response. After decades of research and early trials beginning in 1996, these efforts culminated in 2010 with the approval of the first DC vaccine (Sipuleucel-T) for metastatic castration-resistant prostate cancer, which remains the only therapy of its class approved to date (7, 8). This delayed clinical implementation reflects the challenges faced by DC vaccines, particularly those arising from their limited therapeutic efficacy within immunosuppressive tumor microenvironments (TME), underscoring the need for further research to develop strategies that overcome these limitations while also identifying novel tumor-specific antigens to effectively arm DCs. In this context, this Research Topic features the work from Lee et al., who explored the use of tumor lysate-pulsed DCs in combination with tyrosine kinase inhibitors (TKIs) for the treatment of post-transplant hepatocellular carcinoma recurrence. Their DC-based approach achieved disease control in the majority of patients and nearly doubled survival compared with TKIs alone, offering a promising therapeutic option for a highly vulnerable population with very limited treatment alternatives.

TIL-based therapies emerged from the landmark studies by Prof. Steven Rosenberg and colleagues in the 1980s, which demonstrated that TILs could be isolated directly from tumor tissue, expanded *ex vivo*, and reinfused into patients to achieve effective immune-mediated antitumor responses (9). Over the ensuing decades, this technology has been refined, culminating last year with the approval of the first TIL-based therapy (Lifileucel) for the treatment of unresectable and metastatic melanoma (8). To delve deeper into this topic, Kraja et al. provide in this Research Topic a comprehensive overview of TIL-based therapies, highlighting their potential as both biomarkers and therapeutic agents, as well as the factors influencing their antitumor efficacy, with particular emphasis on how preclinical models and emerging technologies can advance our understanding of TIL function and guide the development of more effective therapies.

CIK cells were first described in the early 1990s by Prof. Ingo Schmidt-Wolf and colleagues, who demonstrated in mouse models that cytokine stimulation of peripheral blood mononuclear cells (PBMCs) *ex vivo* generates a highly cytotoxic cell population able to mount strong antitumor responses *in vivo*. Further characterization revealed that this population is heterogeneous, comprising T cells, NK cells, and NKT cells, which exert tumor-killing activity independently of the major histocompatibility complex (MHC) (3). This approach forms the foundation of CIK-based therapies, which are currently under clinical investigation and have shown encouraging results in specific conditions. In this Research Topic, Yang et al. further investigate the therapeutic potential of CIK cells in preclinical gastric cancer murine models, evaluating the combination of this therapy with chemotherapy and immune checkpoint blockade. In this setting, the authors demonstrate that CIK cells actively home to the tumors and significantly inhibit their growth when paired with chemotherapy, with the addition of immune checkpoint blockade further enhancing this effect. These results illustrate how integrating CIK cell-based immunotherapy with complementary treatment strategies may improve therapeutic outcomes.

Beyond the therapies discuss so far, in recent years, the field has experienced a new revolution with the advent of CARs, which enable the genetic engineering of immune cells to specifically recognize and attack cancer cells, opening a powerful new frontier in immunotherapy. The pioneering work of Prof. Carl June led to the development of the first CAR-T cell therapy to receive approval in 2017 (Tisagenlecleucel), with five additional approvals granted in the following years that have transformed the treatment of B cell malignancies (10, 11). In this Research Topic, five review articles provide a comprehensive overview of the current state-of-the-art, each focusing on different aspects of CAR-T therapies and their application across various clinical settings. First, Tomai et al. trace the evolution of CAR-T cell therapies, showing how early challenges in activation and persistence led to the development of five successive CAR generations and highlighting strategies to improve both preclinical evaluation and clinical translation. Focusing specifically on hematologic malignancies, Liu et al. review recent advances in the application of CAR-T therapies to the treatment of acute myeloid leukemia, which have shown encouraging results in preclinical and early clinical studies, while Morgan et al. highlight the potential of CAR-T therapies as a promising approach for high-risk plasma cell dyscrasias, emphasizing the need to optimize target specificity. Finally, the reviews of Dong et al. and Khan et al. address a critical hurdle in the field: the challenges of applying CAR-T strategies to solid tumors. In these malignancies, CAR-T cells must navigate dense fibrotic stroma, abnormal vasculature, and the hypoxic and acidic conditions of the TME to reach their target cells, while their antitumor activity is often limited by local immunosuppressive signals and heterogeneous antigen expression that facilitate immune evasion (12). Moreover, adverse events such as cytokine release syndrome (CRS) and immune effector cell-associated neurotoxicity syndrome (ICANS) are common in CAR-T-treated patients and, although not exclusive to solid tumors, further shift the risk-benefit balance of CAR-T therapies in these patients (13). These critical issues are thoroughly discussed by Dong et al., alongside with other safety concerns such as acute off-target toxicities in major organs and potential long-term effects, including cytokine-associated hematotoxicity and secondary malignancies. The authors further emphasize the need to reduce therapy-related risks through careful target antigen selection and/or combinatorial strategies, while also highlighting the importance of long-term monitoring to manage potential complications, with patient safety and quality of life as top priorities. Building on this concept, Khan et al. delve deeper into antigen selection strategies and the use of combinatorial antigen-sensing circuits that can shape the therapeutic success of CAR-T cells while reducing off-target effects. The authors also discuss innovative approaches to enhance tumor infiltration and maintain CAR-T cell function within the immunosuppressive TME, including cytokine-armored CARs, protease-regulated CARs, and CARs engineered with chemokine receptors. These two review articles are further complemented with the original research of Aniogo et al., who investigated the use of CAR-T cells targeting carcinoembryonic antigen (CEA) in a murine model of metastatic triple-negative breast cancer. The authors demonstrated that combining this therapy with image-guided radiation therapy (IGRT) enhanced CAR-T cell tumor infiltration and persistence, leading to a significant reduction in both lung metastases and primary tumor burden. This approach highlights a potential strategy to overcome some of the barriers that have limited the effectiveness of CAR-T cells in solid tumors.

In addition to the challenges already discussed, the wider application of CAR-T therapies is further constrained by the technical complexity of their production, as these cells are typically generated *ad hoc* from patient-derived T cells, rendering the process logistically demanding, time-consuming, and costly (14). These limitations have led to growing interest in the development of safer, more accessible, and scalable alternatives to CAR-T cells, particularly those that can be manufactured as off-the-shelf products. In this context, recent advances have extended CAR engineering to other immune cell types, such as NK cells and macrophages. CAR-NK therapies combine targeted antigen recognition with the innate cytotoxicity and favorable safety profile of NK cells, whereas CAR-M therapies exploit the phagocytic and immunomodulatory capacities of macrophages, offering an alternative to direct cytotoxic approaches through a dual mechanism that couples targeted tumor cell clearance with modulation of the TME. Moreover, unlike CAR-T cells, CAR-NK and CAR-M therapies can be derived from allogeneic sources, allowing off-the-shelf production that greatly reduces manufacturing time and cost while improving scalability and accessibility. Although these strategies remain under clinical evaluation and have not yet received regulatory approval for any indication, they represent promising alternatives to CAR-T cell therapies. These and other aspects of CAR-NK and CAR-M therapies are discussed in this Research Topic by dos Reis et al. and Morva et al., respectively. dos Reis et al. highlight the key role of tumor infiltration dynamics and adaptation to the TME in shaping CAR-NK cell efficacy, emphasizing the value of multi-omics approaches in guiding future efforts to improve therapeutic performance. Complementing this perspective, Morva et al. focus on the application of CAR-M therapies for the treatment of solid tumors, leveraging the natural tissue-infiltrating ability of macrophages, and discuss preclinical and early clinical findings alongside the challenges that must be overcome for broader implementation.

Beyond CARs, TCR engineering has emerged as an additional strategy to redirect T cell activity toward tumor cells. While initially overshadowed by the success of CAR-T cells in hematologic malignancies, engineered TCR T cell (TCR-T) therapies are gaining renewed interest, fueled by evidence suggesting they may be better suited to target solid tumors (5, 15). While both CAR-T and TCR-T cells are designed to target tumor-associated antigens, the way they recognize these antigens differs, with CAR-T cells binding them in an MHC-independent manner, whereas TCR-T cells recognizing them only when presented by the MHC (5, 15). This difference carries significant implications for their respective mechanisms of action and therapeutic potential. CAR-T cells target only antigens that the tumor cells express on their surface, often displaying thousands of copies that enable efficient recognition and killing. However, in solid tumors, this high antigen density can limit efficacy, as CAR-T cells tend to remain attached at the tumor periphery rather than fully infiltrating the tissue (5, 15). In contrast, TCR-T cells can target virtually any intracellular or extracellular protein as long as it is presented by the MHC, greatly expanding the pool of potential therapeutic targets. This enables the design of TCRs specific to tumor-restricted antigens, such as neoantigens derived from somatic mutations. Moreover, since MHC-presented antigens are displayed at far lower copy numbers than membrane-expressed antigens, TCR-T cells are less prone to remain confined to the tumor surface, potentially enabling deeper tumor penetration (5, 15). Aiming to provide new tools for TCR engineering, Lennerz et al. present in this Research Topic an innovative antigen-agnostic approach to identify tumor-specific TCRs in solid cancers. The authors compared the TCR repertoires of TILs and adjacent tissue-resident lymphocytes from non-small cell lung cancer (NSCLC) patients, enabling the identification of potential tumor-specific TCR candidates. Four of these TCRs were then engineered into TCR-T cells and tested against both patient-derived tumor cells and NSCLC cells lines, demonstrating effective targeting. Further characterization revealed that three of these TCR candidates specifically recognized a peptide containing the recurrent oncogenic Q61H substitution in KRAS, providing evidence of the potential of this method to detect TCRs recognizing tumor-specific neoantigens.

To complete this journey through the evolving landscape of the field, this Research Topic features the contribution of Looi et al., who provide a comprehensive review of the approaches discussed here, with a particular focus on their current and potential application in the clinical management of nasopharyngeal cancer.

Together, the articles outlined here highlight both the promise and the challenges of implementing cell-based immunotherapies in clinical settings. By enabling the customization of these treatments and their combination with other therapeutic strategies, cell-based immunotherapies offer the potential for highly targeted and versatile cancer management. However, significant barriers to their effective application remain and must be addressed by future research to broaden patient access. To this end, preclinical studies employing advanced *in vitro* and *in vivo* experimental models, combined with the integration of cutting-edge technologies such as novel analytical tools and multi-omics approaches, will be crucial. The development of next-generation cell-based immunotherapies will rely on research conducted using these innovative platforms, which will be instrumental in accelerating the translation of discoveries from bench to bedside.

This Research Topic has been spearheaded by the IMMUNO-model COST Action (CA21135), a collaborative scientific network devoted to fostering innovation in preclinical immuno-oncology models with the ultimate goal of advancing in the treatment of cancer patients by improving their outcomes and quality of life. Within this initiative, IMMUNO-model members have contributed four comprehensive reviews led by Kraja et al., Tomai et al., dos Reis et al. and Morva et al.. IMMUNO-model currently brings together over 400 researchers in basic, preclinical, and clinical cancer immunotherapy across Europe and beyond, representing both academia and industry. Since its establishment in 2022, the network has driven the creation of the IMMUNO-model Knowledge Hub, a dedicated platform for collecting, sharing, and disseminating protocols relevant to immuno-oncology research. IMMUNO-model also actively fosters networking and training initiatives and offers mobility grants to support attendance at key conferences or short-term scientific missions. Now entering the final year of the project, IMMUNO-model continues to welcome participants from around the world to engage in its upcoming activities.
